# Challenging Diagnosis of Invasive Sinus Aspergillosis Mimicking Gradenigo’s Syndrome in an Elderly Patient with T-Cell Lymphoma

**DOI:** 10.3390/geriatrics9010004

**Published:** 2023-12-28

**Authors:** Victoria Ramos de Ascanio, Gloria Liaño-Esteso, David Roldán, Teresa Collazo-Lorduy, Sara Martínez-Flores, José Ángel Hernández-Rivas, Isabel González-Gascón-y-Marín

**Affiliations:** 1 Hematology Department, Hospital Universitario Infanta Leonor, 28031 Madrid, Spain; 2 Radiology Department, Clínica Universidad de Navarra, 28031 Madrid, Spain; 3 Pathology Department, Hospital Universitario Infanta Leonor, 28031 Madrid, Spain; 4 Otorhinolaryngology Department, Hospital Universitario Infanta Leonor, 28031 Madrid, Spain; 5 Geriatrics Department, Hospital Universitario Infanta Leonor, 28031 Madrid, Spain

**Keywords:** T-cell lymphoma, invasive aspergillosis, fungal infection, immunosuppression, elderly

## Abstract

(1) Background: Gradenigo’s Syndrome (GS) is a rare complication of acute otitis media characterized by the triad of diplopia, otitis, and facial pain. The widespread use of antibiotics has significantly reduced its occurrence. (2) Case summary: We present the case of an elderly patient with T-cell lymphoma who developed neurological deficits resembling GS. The patient was ultimately diagnosed with invasive sinus aspergillosis. The diagnostic process was challenging due to the atypical clinical presentation and the lack of specific imaging findings. A biopsy was the most important test for clarifying the diagnosis. (3) Conclusions: The prognosis for this complication is extremely poor without surgery, and the patient died despite adequate antifungal coverage. Therefore, maintaining high clinical suspicion is paramount to avoid adverse outcomes in similar cases, particularly in the geriatric population, wherein this syndrome’s occurrence may not be expected.

## 1. Introduction

In the geriatric population, infections pose a significant challenge due to age-related changes in immune function and the presence of comorbidities. This becomes especially pertinent when examining conditions like Gradenigo’s syndrome (GS). This syndrome is defined by the classic triad of suppurative otitis media (OM), diplopia caused by cranial nerve VI (abducens) palsy and facial pain in the distribution of the trigeminal nerve [1]. Historically, GS has predominantly affected the pediatric population, with bacterial infections serving as the predominant etiological factor. However, with the widespread availability of antibiotics, this complication has become less frequent in children, and there has been a shift in occurrence towards adult patients, though it remains exceptional among the elderly [2]. GS typically arises from contiguous inflammation of the petrous apex of the temporal bone but may also result as an extension of acute sinusitis [2]. The classical triad of symptoms is observed in only a fraction of cases, ranging from 20% to 45% in reported series [3,4]. This variability in presentation has led to confusion surrounding the use of the term GS. To address this issue, McLaren et al. proposed classifying GS into three categories: classic GS (the triad of symptoms with evidence of PA), incomplete GS (two of the triad of symptoms, including abducens nerve palsy with PA), and GS mimics (two or three of the triad symptoms without evidence of PA). In the context of Gradenigo mimics, tumoral or thrombotic etiologies predominate [3].

In older patients, GS is infrequent and insidious, and its unexpected clinical presentation complicates medical management. Age-related decline in immune function and the presence of associated comorbidities make geriatric patients more susceptible to infections and severe complications [5]. In addition, the complex nature of geriatric care highlights the importance of a comprehensive geriatric assessment. This assessment considers not only the underlying condition but also a patient’s overall health, functional status, and comorbidities. It requires a holistic approach to deliver tailored treatment and support to elderly patients, ultimately enhancing their overall quality of life.

This case report describes an elderly patient with T-cell lymphoma who developed neurological deficits resembling GS, ultimately being diagnosed with invasive sinus aspergillosis. The unique aspect of this case lies in the intersection of geriatric care, infectious diseases, and lymphoma management. By sharing this case, our goal is to help raise awareness among physicians about this rare presentation. Early clinical suspicion and the recognition of atypical manifestations are crucial in facilitating timely diagnosis and effective management in similar cases. This report serves as a valuable resource for healthcare professionals who may encounter patients with similar clinical complexities, ultimately promoting better patient care and outcomes.

## 2. Case Description

An 81-year-old female was admitted to the hospital following the diagnosis of peripheral T-cell lymphoma, not otherwise specified (PTCL, NOS), of stage IVB in November 2020. Her previous medical history included arterial hypertension, dyslipidemia, an aneurysm of the interatrial septum, and insulin-dependent diabetes mellitus with inadequate glycemic control.

**Figure 1:**
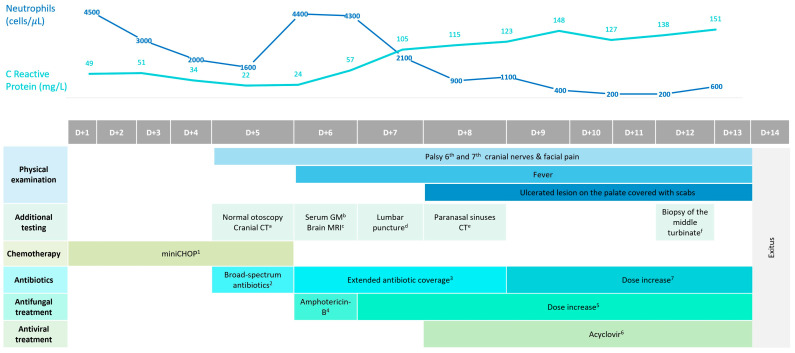
Gantt chart showing the timeline of the clinical, diagnostic, and treatment evolution of the case, as well as the evolution of the laboratory parameters C-Reactive Protein and neutrophil count. ^1^ Cyclophosphamide (400 mg/m^2^) on day 1; Doxorubicin (25 mg/m^2^) on day 1; Vincristine (1 mg) on day 1; Prednisone (40 mg/m^2^) on days 1, 2, 3, 4, and 5. ^2^ Meropenem (1 g) administered every 8 h IV. ^3^ Treatment with meropenem was combined with linezolid at a dose of 600 mg administered every 12 h IV. ^4^ Treatment was initiated with liposomal preparation of amphotericin-B at a dose of 3 mg/kg administered IV. ^5^ Amphotericin-B dose was increased to 5 mg/kg IV. ^6^ Treatment was associated with Acyclovir at a dose of 500 mg administered every 8 h orally. ^7^ Meropenem dose was increased to 2 g administered every 8 h IV. ^a^ Cranial CT: inflammation of the maxillar and sphenoid-ethmoidal sinuses. ^b^ Serum GM (galactomannan antigen test): 0.34 (negative) ^c^ Brain MRI: consistent with CT findings, without identifying PA. ^d^ Lumbar puncture: clear and sterile cerebrospinal fluid. Normal lymphocyte immunophenotype performed via FCM. ^e^ Paranasal sinuses CT: worsening inflammatory changes, with no evidence of bone erosions. ^f^ Biopsy of the middle turbinate: septate hyphae compatible with *Aspergillus* spp. infection.

After a comprehensive geriatric assessment (CGA), the patient was categorized as fit. CGA was performed by a specialized geriatrician who also made recommendations on treatment intensity and implemented nutritional and physical interventions. Geriatric domains were assessed using the following scales: G8 (screening), Charlson (comorbidities), Barthel (activities of daily living), Lawton (instrumental activities of daily living), SPPB and gait speed (physical function), Fried (frailty), mini-mental (cognition), GDS-SF (mood), MNA-SF (nutrition), and polypharmacy (≥5 medications). Thus, an initial cycle of mini-CHOP chemotherapy was administered (cyclophosphamide: 400 mg/m^2^; doxorubicin: 25 mg/m^2^; vincristine: 1 mg total dose; and prednisone 40 mg/m^2^). Five days later, the patient began to experience acute and severe hemifacial pain accompanied by diplopia. Figure 1 represents a schematic timeline of the clinical evolution of the case, the diagnostic tests conducted, and the therapeutic management of the patient.

**Figure 2:**
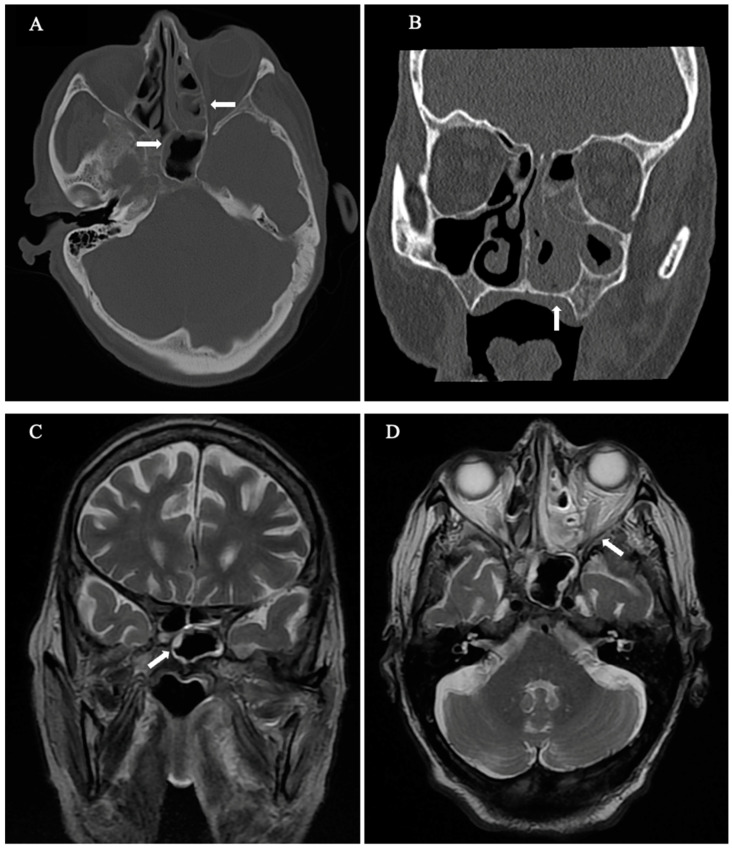
(**A**–**D**) CT and MRI scans show an inflammation of the paranasal sinuses without erosion of the petrous bone (note that bone destruction in these cases can be extensive, subtle, or even inapparent). (**A**) Axial CT: Moderate to severe mucoperiosteal thickening predominantly in the posterior ethmoidal cells, with subtotal cell occupancy (horizontal arrows). (**B**) Coronal CT: Subtotal occupation with mucoperiosteal thickening and soft tissue attenuation in ethmoidal and maxillary sinuses as well as in the left nasal cavity (vertical arrow). (**C**) Coronal T2 WI MRI: Mild hyperintense mucosal peripheral thickening of the sphenoid sinus (diagonal arrow). Note central hypointensity, which might be due to either air or fungal elements. (**D**) Axial T2 WI MRI: hyperintense peripheral soft tissue in the left paranasal sinuses with central hypointensity within the posterior ethmoidal cells, consistent with fungal elements (diagonal arrow). No fat stranding indicating invasion outside the sinus.

Neurological physical examination suggested palsy of the sixth and seventh cranial nerves (CN), along with numbness in the distribution of the fifth CN (V1). At that time, no fever or other symptoms were detected, and the patient’s blood tests did not indicate neutropenia (4.4 × 10^3^/μL). However, the levels of serum inflammatory markers such as C-reactive protein (CRP) began to increase, as depicted in Figure 1. The otoscopy results were normal, and an urgent cranial computed tomography (CT) scan detected inflammation of the maxillary and sphenoethmoidal sinuses. The brain magnetic resonance imaging (MRI) results confirmed the findings of the CT without identifying PA (Figure 2A–D). The serum galactomannan antigen test was negative, with a value of 0.34, and the beta-D-glucan test was not available at our center.

Considering the focal neurological deficits and the oncohematological diagnosis, a lumbar puncture was performed, revealing cerebrospinal fluid that was clear, sterile, and without abnormalities, including a normal lymphocyte immunophenotype determined via high-flow cytometry. Despite these findings, broad-spectrum intravenous (IV) antibiotics were started.

The following day, the patient developed fever and neutropenia, so a treatment with a liposomal formulation of amphotericin-B administered at a dose of 3 mg/kg IV along with high doses of IV acyclovir was started. Unfortunately, her symptoms progressed with the appearance of an ulcerated lesion on the palate covered with scabs and the association of IX CN palsy. Additionally, her clinical condition deteriorated, presenting a cough, expectoration, a decreased level of consciousness, and a continuous fever.

**Figure 3:**
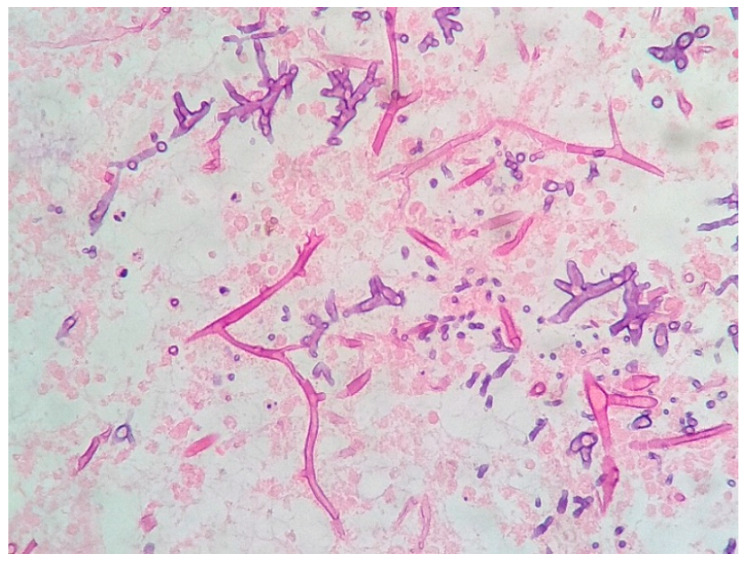
Histopathology of the middle turbinate biopsy showing septate hyphae (Hematoxylin-eosin stain, ×400).

A new CT scan of the paranasal sinuses was requested, showing worsening inflammatory changes, with no evidence of bone erosions. Following these findings, a biopsy of the middle turbinate was performed, and histological examination revealed several septate hyphae that were compatible with an *Aspergillus* spp. infection (Figure 3), confirming the diagnosis of a proven invasive aspergillus infection. Regardless of proper antifungal coverage, the day after the histological diagnosis, the patient died due to this infectious complication.

## 3. Discussion

Invasive paranasal sinus aspergillosis is a rare fatal condition mostly observed in immunocompromised patients. Moreover, the early diagnosis of this entity is a challenge due to the heterogeneity of the clinical manifestations [6]. To the best of our knowledge, this is the first published case of an elderly patient with T-cell lymphoma complicated by invasive sinus aspergillosis mimicking GS.

CGA holds immense significance in the management of the elderly lymphoma population. Prior to initiating treatment, conducting a CGA allows for a thorough evaluation of a patient’s overall health status, functional capacity, and individual vulnerabilities [7]. In our case, despite the patient being considered fit for treatment, her advanced age prompted a decision to initiate a reduced-intensity treatment provided at 50% of the standard regimen (mini-CHOP). However, it is important to note that even with this tailored approach, the patient encountered an infectious complication, possibly influenced by her pre-existing diabetes [8]. This emphasizes the multifaceted nature of geriatric care, where the consideration of comorbidities and individual patient factors becomes crucial in optimizing treatment outcomes.

In the present case, the diagnosis of aspergillosis was difficult not only because it is rare to find this germ in “Gradenigo’s-like” syndrome but also because the lymphoma itself was included in the differential diagnosis. Liu et al. carried out a review of cases of GS published from 1980 to 2021, collecting 60 publications, of which only one was due to invasive aspergillosis, with bacterial infections being the most frequent etiology [9]. On the other hand, McLaren et al. published 41 cases of GS mimics, and in this series, most underlying etiologies were thrombotic or neoplastic, including a case of a T-cell lymphoma [3].

Invasive fungal sphenoid sinusitis is often misdiagnosed, allowing the progression of the infection and delaying treatment [10]. Our patient was diabetic and had received a course of chemotherapy with corticosteroids in addition to the immunosuppression associated with an aggressive lymphoma. All of these are considered risk factors for invasive aspergillosis. However, the lack of initial fever and neutropenia, as well as the onset of neurological symptoms, led us to rule out other diagnostic possibilities and to delay the prompt initiation of antifungal treatment. The elevation of acute-phase reaction parameters in an immunocompromised host may help distinguish a fungal infection from other Gradenigo mimics such as cancer or thrombotic etiologies.

Another difficulty was the absence of bone involvement in the CT and MRI results. This finding is rare in incomplete GS but may be present in cases of mimics [3]. In addition, in the early stages of invasive sphenoid sinus aspergillosis, CT and MR imaging are non-specific [11,12,13]. The exact route by which this fungal pathogen reaches the apex of the paranasal sinus remains unclear [14]. It is speculated that this may occur through the connection of the base of the paranasal sinus with the apex, through vascular channels or fascial planes [15]. In our case, paranasal sinus involvement was by contiguity, which is also unusual.

The standard treatment for confirmed invasive aspergillosis typically involves a combination of surgery and antifungal medications. In the case of our patient, surgery was not feasible due to her deteriorating clinical condition, even though she had initially been deemed a fit candidate before undergoing lymphoma treatment. Regrettably, her clinical condition worsened rapidly, despite the administration of empirical antifungal treatment. The choice of antifungal therapy varies depending on whether a diagnosis of invasive aspergillosis has been verified or other invasive molds are also suspected. If a diagnosis of invasive aspergillosis has been established, voriconazole or isavuconazole are the agents of choice. On the other hand, empirical treatment with liposomal amphotericin-B provides antifungal activity against both *Aspergillus* spp. and other molds such as Mucorales, so it should be used if the fungus involved in the infection has not yet been confirmed, as in the case described [16]. It is noteworthy, however, that for optimal efficacy against Mucorales, higher doses of liposomal amphotericin-B, typically in the range of 5–10 mg/kg/d, are recommended, contrasting with the lower doses used for certain other invasive fungal infections.

## 4. Conclusions

This case report highlights the complexity of diagnosing and treating invasive sinus aspergillosis in an elderly patient with T-cell lymphoma. It contributes significantly to the existing knowledge in several ways. Firstly, it highlights the importance of considering invasive aspergillosis as a differential diagnosis in immunosuppressed patients who present with neurological symptoms that mimic GS, even when imaging tools do not reveal the presence of PA. Secondly, it underscores the necessity of promptly performing a biopsy in such cases to accurately diagnose the patient and initiate appropriate treatment. Thirdly, it emphasizes the significance of administering specific antifungal therapy and/or surgical debridement in the management of these patients. This case underscores the critical need for a high index of suspicion, thorough evaluation, and timely intervention in similar clinical scenarios to improve patient outcomes.

## Data Availability

The data presented in this study are available on request from the corresponding author.
